# Erythema nodosum masking nephrogenic systemic fibrosis as initial skin manifestation

**DOI:** 10.1186/s12882-017-0666-7

**Published:** 2017-07-24

**Authors:** Kar Wah Fuah, Christopher Thiam Seong Lim

**Affiliations:** 1 Department of Medicine, Serdang Hospital, Serdang, Malaysia; 2 Unit of Nephrology, Department of Medicine, Faculty of Medicine and Health Sciences, Universiti Putra Malaysia, Serdang, Selangor Malaysia

**Keywords:** Nephrogenic systemic fibrosis, Gadolinium, Erythema nodosum

## Abstract

**Background:**

Nephrogenic systemic fibrosis (NSF) is a complication of the gadolinium-based contrast agent used in imaging studies. It is typically characterised by hard, erythematous and indurated skin plaques with surrounding subcutaneous oedema. Distinct papules and subcutaneous nodules can also be seen. Fibrocytes in NSF are immunohistochemically positive for CD34.

**Case presentation:**

We present a case of NSF occurred after gadolinium exposure in which the initial presentation mimics an erythema nodosum (EN)-like picture. An initial skin biopsy showed EN. Subsequently the patient developed progressive skin and joints contracture. A repeated skin biopsy done three months later confirmed the diagnosis of NSF. As far as we are aware, this is the second reported case of NSF that mimicked the presentation of EN in the early phase of the disease.

**Conclusions:**

The appearance of EN-like disease can be one of the early manifestations of NSF. We hope that early recognition of this unusual presentation can alert the physician or nephrologist to the potential diagnosis of NSF.

## Background

NSF is a fibrosing cutaneous disorder that has been increasingly recognized to have systemic manifestations. In the majority of NSF patients, symptomatic skin involvement is the initial presentation of the disease. Affected individuals may experience skin discoloration, induration, itchiness, ache, paraesthesia or burning sensation confined to the lower limbs. This will be followed by hardening and thickening of the skin of the extremities and trunk, often resulting in flexion contractures [1]. Histologically NSF is characterised by an increased in spindle-shaped dermal cells, with CD 34 positive and procollagen I fibrocytes deposition in the dermis. This dual positivity (CD34 and procollagen I) is characteristic of the so-called “circulating fibrocytes,” of bone marrow origin [2]. The common link to all patients developing NSF is the history of renal impairment and gadolinium-based contrast agent (GBCA) exposure. We present here a case whereby the initial presentation of NSF mimics the skin histology of erythema nodosum before manifesting with all the usual clinical and histopathological features of NSF.

## Case presentation

**Fig. 1:**
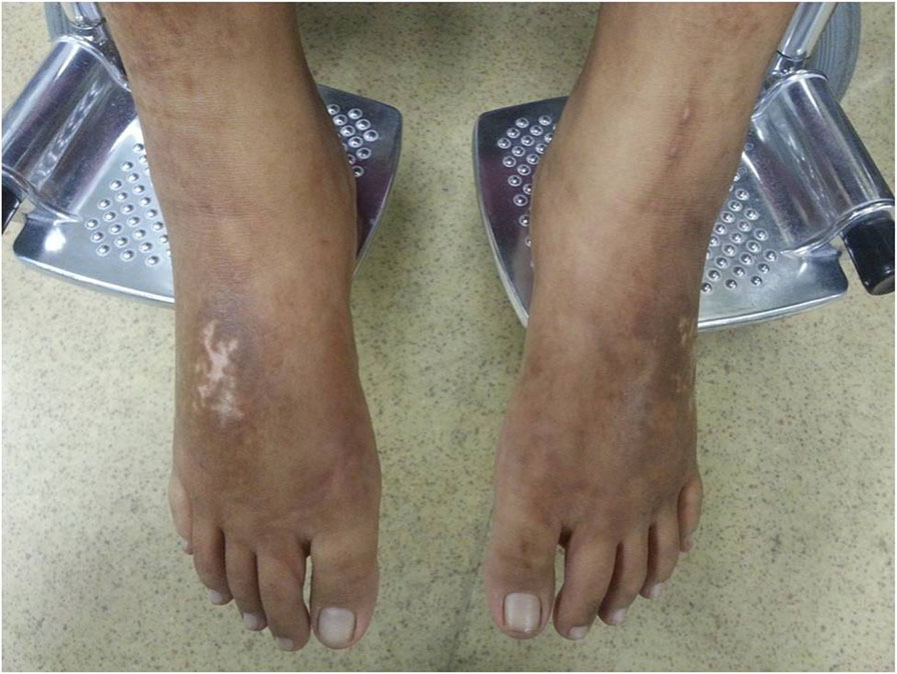
Bilateral ankle odema and dusky dermal plaques

**Fig. 2:**
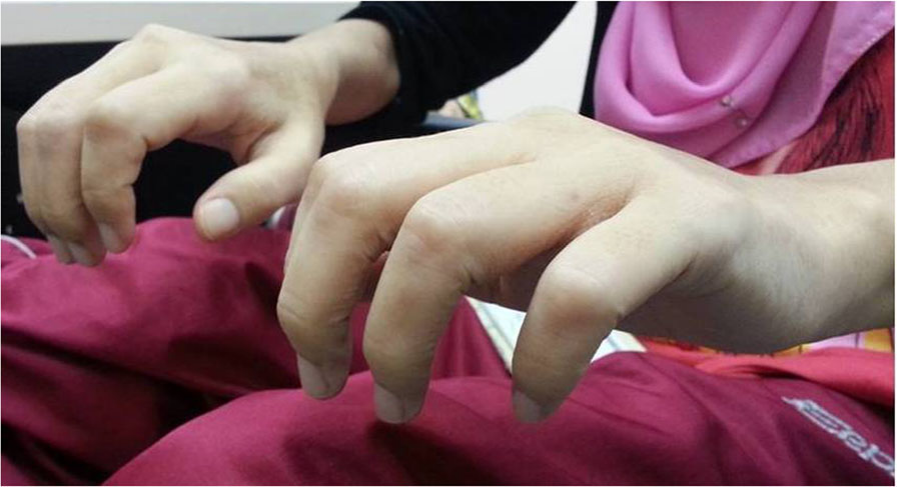
Tightening of skin and contracture of the hands

The patient was a 23-year-old lady with end stage renal failure (ESRF) secondary to glomerulonephritis. She had been receiving automated peritoneal dialysis (APD) for 5 years before presenting to the emergency department with an asymptomatic right sided clavicular lump. The lump was treated conservatively by the attending emergency physician. During one of the subsequent APD clinic follow-ups, she again raised the concerns of the persistent right clavicular lump. She was referred to the orthopaedic surgeon for a second clinical opinion. Ultrasound scan of the clavicle was carried out. It reported the lump as an isoechoic lesion in which the possibility of an abscess formation cannot be ruled out. In order to further clarify the nature of the lump, a Magnetic Resonance Imaging (MRI) scan with gadodiamide (Omniscan, GE Health Diagnostics, Amersham, UK) at a dose of 0.1 ml/kg was carried out. 2 days after the MRI scan, she experienced a febrile illness which lasted for more than two weeks. The illness was associated with migratory joint pains, a feeling of malaise and fatigue. Concurrently, she was noted to have multiple dusky erythematous nodules over her lower limbs with corresponding pitting edema up until the ankles (Fig. 1). A skin biopsy of the lesion revealed erythema nodusum (EN) in which the lymphocytes and histocytes were reported to have infiltrated the subcutaneous layers, mainly involving the fibro adipose septa and also the lower part of the dermis. There was also necrosis of the adipose tissues. Perivascular lymphocytic infiltration was seen in the upper dermis. Blood chemistry panel showed moderate aneamia without leukocytosis. Rheumatic fever screening, Anti-Streptomycin O Titre (ASOT) and mycoplasma antibody tests were negative. Similarly, the echocardiogram showed good ejection fraction without any vegetation or valvular lesion. She was provisionally treated as having possible streptococcal infection with EN. She responded well with intravenous antibiotic and subsequently discharged well. However, three months later, she complained of gradual skin tightening over her fingers, dorsum of the hands and feet (Fig. 2). Soon she became semi wheelchair-bound due to the worsening contracture of her joints. A repeated skin biopsy over her shin showed changes compatible with NSF with CD 34 positive cells in the dermis. The diagnosis was explained to the patient and family. A permanent catheter was inserted and she was started on hybrid therapy (APD supplemented by twice per week high-flux hemodialysis). Her Kt/V improved (2.25 to 2.94) after a period of intensive hybrid therapy. A trial of low dose prednisolone was initiated and she was referred to occupational and physiotherapist for rehabilitation of her stiff joints.

Unfortunately, despite dialysis treatment intensification, she succumbed to cardiac arrhythmia.

## Discussion

Nephrogenic systemic fibrosis (NSF) is a progressive, potentially debilitating multi-organ system fibrosing disease related to exposure of the gadolinium-based contrast agents (GBCA) used in magnetic resonance imaging in patient with kidney failure [1]. NSF affects patients in advanced stage of chronic kidney disease (CKD).

Clinically, NSF varies in its manifestations from patient to patient and over time. Based on a NSF literature encompassing a total of 130 reported patients, the earliest lesions frequently appeared in the lower extremities (85%), followed by the upper extremities (66%) and the trunk (23%) [3]. The skin involvement is often symmetrical and bilateral. The usual initial presenting features were skin discoloration, induration, itchiness, ache and non-specific neuropathic symptoms confined to the lower limbs. Insomnia and transient, diffuse alopecia has also been described in the early stages. The predominant symptom at the later stage was symmetrical skin stiffness of the extremities with or without restricted joint motion [1].

NSF was previously known as nephrogenic fibrosing dermopathy (NFD) and dialysis-associated systemic fibrosis [4]. It was originally named NFD since it was originally thought that the lesions were limited to the skin, it is now well documented that these lesions extend beyond the dermis and can involve joints, deeper structures such as muscle, lungs and heart.

The risk of developing NSF in a patient with severe kidney impairment following exposure to GBCA is roughly 4% regardless of gender, race or age [5]. The onset between initial exposure to the GBCAs and the diagnosis of NSF can vary between 2 months to 18 months. Acute kidney injury, thromboembolic event, infection, malignancy and high dose erythropoietin usage are some of the risk factors that predisposed to NSF. Increasing cumulative gadodiamide exposure, high dose epoetin-b treatment, and higher serum concentrations of ionized calcium and phosphate increase the risk of gadodiamide-related NSF in CKD patients [1].

Gadodiamide has a half-life of 1.3 h in a healthy person. The half-life will increase to 10 h in patient with CKD stage 3b with estimated glomerular filtration rate (eGFR) of 20 to 40 mL/min, and this will further prolong to 34 h in patients with end-stage renal disease (ESRD). For patients with ESRD, the half-life is reduced markedly to between 1.9 to 2.6 h if haemodialysis procedure is done following the administration of gadolinium [6].

GBCA is made up of Gd3+ ion chelated by a proprietary ligand [7]. Each of the available agents has a small tendency for the Gd3+ ion to separate from its ligand [8]. Free Gd3+ ion is theoretically available to interact with other negatively charged substrates. These free gadolinium ions deposition in the dermis or surrounding tissue will results in tissue inflammation and fibrosis. Hence the risk of NSF increases with the severity of CKD. The risk ranges from 2.5–5% in patients with CKD 5 to virtually unheard of in non-dialysis patients [9]. Patients on peritoneal dialysis (PD) has a higher incidence of NSF when compare to haemodialysis as the rate of clearance of CBGAs are much slower in PD.

EN is thought to be some form of hypersensitivity reaction. It may be idiopathic in origin or associated with underlying systemic diseases or drug therapies. Our patient has post-streptococcal like infection with EN changes demonstrated in the skin histology soon after she was being exposed to gadolinium. In our part of the world, EN is commonly associated with streptococcal infection. However, the findings of EN in the initial skin biopsy could also represent the early manifestation of NSF [10]. In NSF, the skin may be variably affected with subtle, superficial papules and plaques, deeper dermal or subcutaneous induration. The most frequently seen lesions reported were plaques (58%), papules (32%) and nodules (17%) [2]. Similar to ours, another retrospective single centre study has concluded that the risk of developing NSF is markedly increased when there is concurrent infection [11].

After exposure to GBCAs, the time of onset of NSF ranges from a few days to 6 months [12]. In almost all of the cases, NSF is chronic and unremitting in nature. Joint contractures can be so debilitating that patients become wheelchair-bound within days to weeks after onset of the disease [12]. Interestingly, our patient has symptoms of migratory joint paints, fever and malaise soon after being exposed to gadolinium. Although there has not been any report in the literature regarding the occurrence of acute systemic inflammatory reaction after receiving intravenous contrast, we are still incline to think that the patient’s symptoms are sequelae of gadolinium exposure rather than a true EN disease.

In our case, the possible differentials for thickening and hardening of the skin overlying the limbs and trunk involved a variety of other disorders, such as systemic sclerosis (scleroderma), scleromyxedema, eosinophilic fasciitis and calciphylaxis [13]. The temporal history of gadolinium exposure in an advanced CKD patient coupled with tissue histopathological features of NSF can distinguished among them [14].

Unfortunately, there is no effective treatment available to treat NSF. Various trials involving corticosteroid, plasmapheresis, thiosulfate, methotrexate has been tried with no promising results [15]. The mainstay of the management is to provide continuous medical and psychological support to the patient and family. Occupational and physiotherapy to optimize the affected joints functionality are of utmost importance. Avoidance of gadolinium appeared to be the major preventive measure for NSF.

In this case, the administration of GBCA has clearly not benefited the patient. Retrospectively, alternative imaging method should have been considered. Once considered to be safe to be used in the setting of renal disease, the US Food and Drug Administration and the European Medicines Agency has prohibited the use of three types of gadolinium based agents (Magnevist, Omniscan, and Optimark) in the setting of renal diseases in 2010 [16]. Our unfortunate incident happened in early 2012. It is not the intention of this paper to apportion blame as to why gadodiamide is being administered inadvertently but to ensure such episode will not occur again. It could be due to the fact that since no cases of NSF have been reported in our country before, there may be a misconception among the health professionals that GBCAs are safer than contrast agent used in computer tomography. Thus far, various inter departments dialog had ensued and agreement has been made that no GBCAs should be given to patients with moderate to severe kidney disease. The next-of-kind of the patient had been duly informed of the adverse event and updated continuously throughout the incident.

## Conclusion

Our experience with this patient shows that the early skin features of NSF can be histopathologically indistinguishable from EN. Besides, the acute inflammatory like reaction experienced by the patients post gadodiamide has further aroused the suspicion of EN. Nevertheless, we believe that the patient’s presentation is within the spectrum of manifestation of NSF. We hope by highlighting this case, we can avoid the potential pitfall of missing early phase of NSF in CKD patients who received CBGA.
